# Dosimetric effects of rotational errors for single isocenter multiple targets in HyperArc plans: A phantom and retrospective imaging analysis study

**DOI:** 10.1002/acm2.14214

**Published:** 2023-12-15

**Authors:** Samane Golmakani, Andrew N. McGrath, Timothy J. Williams

**Affiliations:** ^1^ W.P. Holman Clinic Royal Hobart Hospital Hobart Tasmania Australia

**Keywords:** dose coverage, dosimetric effect, margin size, rotational errors, SIMT HyperArc

## Abstract

**Purpose:**

This study uses a phantom to investigate the dosimetric impact of rotational setup errors for Single Isocenter Multiple Targets (SIMT) HyperArc plans. Additionally, it evaluates intra‐fractional rotational setup errors in patients treated with Encompass immobilization system.

**Methods:**

The Varian HyperArc system (Varian Medical systems) was used to create plans targeting spherical PTVs with diameters of 5, 10, and 15 mm and with offsets of 1.3–5.3 cm from the isocenter. Dosimetric parameters, including mean and maximum dose, D99% and D95% were evaluated for various rotational setup errors ranging from 0.5° to 2° for the PTVs and certain CTVs created within PTVs. These rotational errors were applied in an order and direction that resulted in the maximum displacement of targets. The rotation was applied both uniformly around all three axes and individually around each axis. Furthermore, to link the findings to actual treatment scenarios, the intra‐fractional rotational setup errors were obtained for stereotactic cranial patients treated with the Encompass system using CBCT images acquired during treatments.

**Results:**

The maximum displacement of 2.7 mm was observed for targets located at 4.4 and 4.5 cm from the isocenter with rotational setup errors of 2°. The dose reduction for D99% values corresponding to this displacement were about 50%, 40%, and 30% for PTVs with diameters of 5, 10, and 15 mm, respectively. Both D99% and D95% showed a consistent trend of dose reduction across various rotational errors and PTV volumes. While the maximum dose remained consistent for different targets with various rotational errors, the mean dose decreased by approximately 25%, 12%, and 6% for PTVs with diameters of 5, 10, and 15 cm, respectively, with rotational errors of 2°. In addition, by analyzing CBCT images, the absolute mean rotational setup errors obtained during treatment with Encompass for pitch, roll, and yaw were 0.17° ± 0.13°, 0.11° ± 0.10°, and 0.12° ± 0.10° respectively. This data, combined with existing studies, suggest that a 0.5° rotational setup error is a safe choice to consider for calculating additional PTV margin to ensure adequate CTV coverage.

Therefore, the assessment of maximum displacement and dosimetric parameters in this study, for a 0.5° rotational error, highlights the need for an additional 0.7 mm PTV margin for targets positioned at distances of 4.4 cm or greater from the isocenter.

**Conclusions:**

For SIMT Plans, a 0.5° rotational setup error is recommended as a basis for calculating additional PTV margins to ensure adequate CTV coverage when using the Encompass system.

## 1 INTRODUCTION

Stereotactic radiotherapy is a highly effective technique for treating brain tumors. Recent advancement such as the integration of automated solutions like HyperArc into the Eclipse treatment planning system (Varian medical systems, Palo Alto, CA), the implementation of purpose made masks for patient positioning, and the utilization of a 6° of freedom (6DOF) treatment couch in conjunction with advanced imaging have significantly enhanced the appeal, accessibility, and efficiency of this treatment technique.

HyperArc is an automated planning and delivery system for cranial stereotactic radiosurgery (SRS) developed by Varian Medical Systems. It uses Volumetric Modulated Arc Therapy (VMAT) delivered from multiple non‐coplanar angles to achieve a highly conformal dose coverage. This can be achieved using a single isocenter with single or multiple targets. This automated system has made simultaneous treatment of multiple brain tumors more accessible with efficiency gains in both plan preparation and treatment delivery.^1^, ^2^, ^3^

However, unlike single isocenter plans with a single target, Single Isocenter Multiple Targets (SIMT) plans are sensitive to rotational set‐up errors.^4^, ^5^, ^6^, ^7^, ^8^ In their retrospective study using patient data treated with HyperArc, Sagawa et al.^9^ investigated the dosimetric impact of setup errors on plans with both single and multiple brain metastases. Their results showed that rotational setup errors for SIMT plans caused non‐negligible underdosage for PTVs whereas the dosimetric parameters for single metastases were found to be comparable with the non‐rotated reference plans.

Ohira et al.^10^ demonstrated that HyperArc plans create higher conformity with rapid dose falloff compared to conventional SRS VMAT plans. As a result rotational errors may have a more considerable impact on the dose coverage of SIMT HyperArc plans particularly for targets located further from isocenter.^9^

Roper et al.^11^ used retrospective patient data to investigate the effect of rotational error on D95% and V95% parameters in VMAT based SRS treatment. This study focused on assessment of only two tumors in each patient. They demonstrated that rotational set‐up errors can compromised the tumor coverage, and smaller PTV located further away from isocenter are more susceptible to these errors.

Nakano et al.^6^ performed a geometrical simulation to evaluate the effect of rotational error on the geometrical coverage of targets with varying volumes at distances ranging from 0 to 15 cm from the isocenter. They determined the maximum distance between the isocenter and the targets that would still maintain a reduction tolerance of 5% for geometrical coverage for each target volume. In order to satisfy the 5% tolerance for all targets, they recommended excluding targets located 7.6 cm away from the isocenter and treating them separately. This study was solely geometrical involving vectors and rotational matrices and did not use any dose calculation for the evaluation of rotational errors.

Chang^12^ developed a statistical model to determine the additional margin required to compensate for the loss of dose coverage caused by rotational setup errors in SIMT plans. This study also used a geometrical approach for determining the statistical model. They concluded that the rotational errors cannot be disregarded in SRS treatments, especially when the distance between the isocenter and target is large.

In their study of optimising the PTV margin for SIMT, Rojas‐Lopez^7^ showed the significance of both the order (X, Y, and Z) and direction (clockwise (CW), counter‐clockwise (CCW)) of rotational errors on displacement of the targets. They observed that for a target positioned 52 mm away from the isocenter, the displacement of the target varied from 0.2 to 3.0 mm based on the specific order and direction of the rotational errors.

These studies collectively highlight the sensitivity of SIMT plans to rotational setup errors, emphasizing the importance of mitigating their effects. Understanding and managing these errors can help to optimize and enhance the overall effectiveness of SIMT treatment. It is important to note that patient setup errors are corrected when detected using image‐guided technology and a 6DOF couch. However, these imaging procedures usually take place at the start, mid, or end of each treatment session. Therefore, certain intrafractional setup errors resulting from patient movement during treatment may remain uncorrected.

In this study, the dosimetric effect of intrafractional rotational setup errors was evaluated using a phantom. Six spherical Planning Target Volumes (PTV) with diameters of 5, 10, and 15 mm were created in a phantom. For each target, the order and direction of rotation that resulted in the maximum displacement was calculated. Then, the dose coverage across plans with different magnitudes of rotational errors was calculated using the specific combination that resulted in the maximum displacement. The dosimetric parameters for different plans as well as different targets within a plan were evaluated and compared.

Through using a phantom a more systematic approach can be adopted incorporating well‐defined volumes, distances from the isocenter, and specific magnitudes of rotational errors. This approach allows for a more controlled and standardized assessment of the effect of rotational errors on various dosimetric parameters. The results of this phantom study were compared with the existing literature which included retrospective patient data analyses, geometric and statistical studies.

Furthermore, the CBCT images acquired during clinical stereotactic cranial treatments were analyzed to estimate intra‐fractional rotational setup errors during treatment. A combination of these findings was used to evaluate the appropriate rotational setup errors for calculating an additional PTV margin.

## 2 METHODS

### 2.1 Phantom study

**FIGURE 1:**
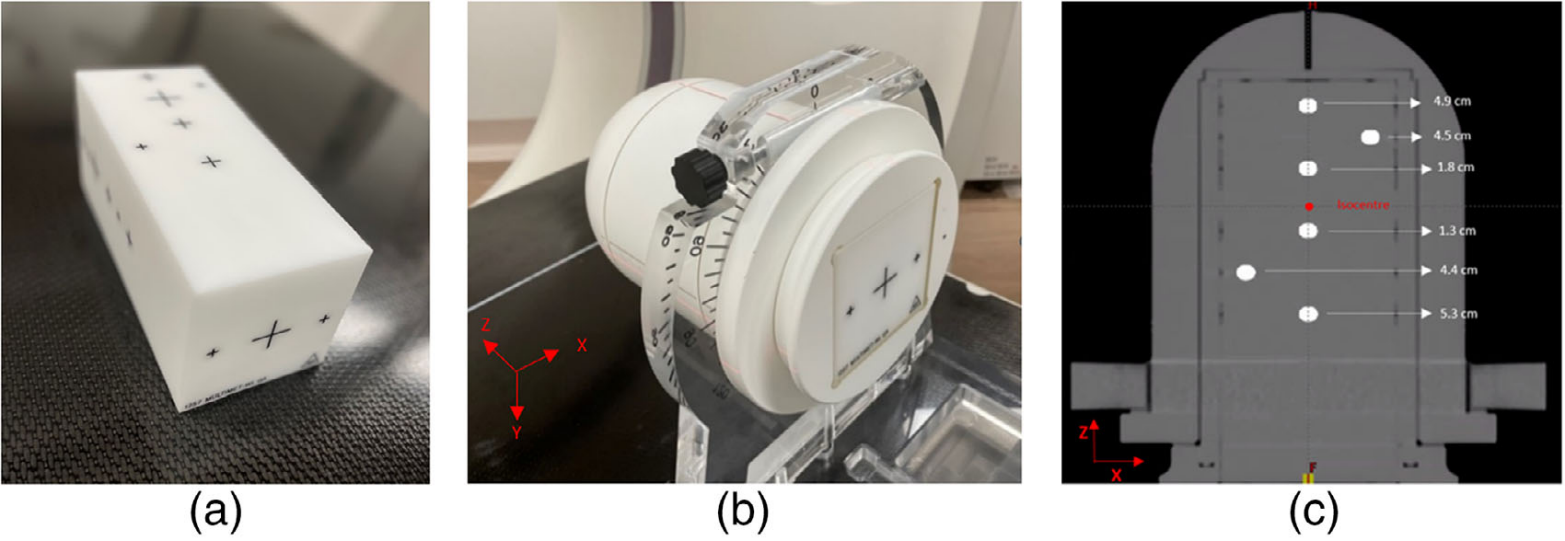
MM‐WL phantom (a) MM‐WL inside StereoPHAN (b) Coronal CT image of MM_WL and StereoPHAN with PTV structures and distances from the isocenter (c).

In this study a StereoPHAN phantom (Sun Nuclear corporation, Melbourne, FL) in conjunction with the MultiMet Winston‐Lutz insert (MM‐WL) (Sun Nuclear Corporation, Melbourne, FL) was used as the basis for creating rotated phantom datasets. The StereoPHAN is a specialized phantom designed for end to end and quality assurance in stereotactic techniques and enables the insertion of a wide range of inserts into the phantom body. The MultiMet Winston‐Lutz insert contains 6 tungsten carbide spheres of 5.0 ± 0.025 mm diameter positioned precisely within the insert with a positional tolerance of ± 0.050 mm. Figure 1 shows the MM‐WL phantom (a) and the configuration of the phantom in this study (b).

The StereoPHAN phantom, with the MM‐WL insert, was scanned with a Canon Aquilion CT scanner following the departmental stereotactic protocol. The scan was performed with a slice thickness of 1 mm, with 512 × 512 pixels and tube voltage of 120 kVp.

The Eclipse Scripting API (ESAPI) was used to create consistent spherical contours with various diameters. The script takes a coordinate defining the centre of the sphere and a value for diameter as inputs. It then creates a spherical structure around the given center with the given diameter. Contours for Clinical Target Volumes (CTVs) with diameters of 8 and 13 mm were created centered on each sphere in the MM‐WL phantom. These contours are referred to as CTV8mm and CTV13mm henceforth. To create PTVs, a 1 mm isotropic margin was added to the CTVs. PTVs with diameters of 10 and 15 mm were created, known as PTV10 mm and PTV15 mm. In addition, PTVs with a 5 mm diameter were created. However, due to the small volume of PTV5mm, no corresponding CTV structures were created.

The 1 mm PTV margin added to the CTV accounts for uncertainties introduced throughout the treatment process such as uncertainties in contouring the targets, mechanical and dosimetry of linear accelerator, patients set up, and more. However, this margin does not address the uncertainties resulted from rotational errors in SIMT plans. An additional margin may be needed to account for these uncertainties.

The phantom mass density was overridden and set to 1.0 g cm^−3^, with water assigned as the material.

An Encompass couch structure model was inserted into each image set. This structure is a requirement for creating HyperArc plans and is used by Eclipse (Varian Medical System, version 15.6) for collision prediction. Three SIMT HyperArc plans were created for the three sets of target diameters, referred to as PTV5 mm, PTV10 mm, and PTV15 mm plans. Each plan was designed to deliver 20 Gy in a single fraction to the six PTV spheres using 6MV flattening filter free (FFF) model (*D*_max_ = 1.3 cm) on a TrueBeam linear accelerator (Varian Medical system) fitted with a millennium MLC.

Automatic Lower Dose Objective (ALDO), which is an integral component of HyperArc in Eclipse optimization, was used for all plans. ALDO automates the attainment of equal relative volumetric coverage of 98% among all targets. If one or more of the targets cannot achieve that goal, it will lower the goal to 97% for all targets. The Acuros algorithm (dose to medium; version 15.6) with a 1 mm grid size was used for dose calculation in these plans. Each plan consisted of three half arcs (180°) and one full arc (360°) with three non‐coplanar couch angles (45°, 315°, and 270°). The encompass couch model was removed from the final plan to eliminate any uncertainty related to its positioning.

The dose coverage for D99% of targets met or exceeded 98% of the prescribed dose in all three plans. These three plans serve as the reference plans and were used for the subsequent evaluation of rotational errors. The isocenter of each plan was placed automatically into the center of mass of the PTVs as part of HyperArc planning process. The isocenter location was the same for the three reference plans. The distance from the isocenter to the center of each target varied from 1.3 to 5.3 cm, as shown in Figure 1c.

To evaluate the effect of the rotational errors, the initial phantom image set was rotated around each three orthogonal axes individually and also then with combined axes rotations from 0.5° to 2° with a 0.5° increment around the isocenter of the plan.

For the combined axes rotations, the order and direction that led to the maximum displacement of target were determined for each target by evaluating the displacement from all 48 possible combinations. The analysis was performed using a C# script. The script used 3D rotation matrices to rotate the coordinate center of each target around different axes, with different orders and directions, and then calculated the displacement point from its initial coordinates. The combination that produced the maximum displacement for each target was defined. While there were multiple combinations that resulted in maximum displacement for each target, the one most frequently observed among the targets was selected. These combinations are represented by specifying the order of rotations around X, Y, and Z axes and their respective directions, either CW or CCW. Ultimately, two combinations were chosen: Z(CCW)Y(CCW)X(CW) for targets located at 4.4 and 4.5 cm from the isocenter, and X(CCW)Y(CCW)Z(CCW) for remaining targets. The image sets were rotated based on these specific combinations, resulting in the creation of two image sets for each plan: one for targets located at 4.4 and 4.5 cm, and another for remaining targets.

In addition, individual rotational errors were introduced in the CCW direction around each axis, and they were denoted by the corresponding rotation axis (X, Y, or Z) along with the magnitude of rotation (e.g., X = 0.5°). The axes have been shown in Figure 1.

The rotation of phantom image was performed with an in‐house C# program that took the original CT dataset and created a new DICOM dataset with the rotations applied. Each voxel value in the new dataset was set by performing a tri‐linear interpolation of the original CT dataset, with the position determined by the rotation around the plan isocenter.

In order to rotate the spherical structures in agreement with the rotation applied to the CT dataset, the central coordinate of all spheres in the reference plan were rotated using 3D rotation matrices. The rotation was based on the combination that led to the maximum displacement of the target center. Then, within the rotated image dataset, ESAPI was used to generate spherical contours around the repositioned points. The magnitude, order and direction of the rotated center point remained consistent with the rotated image dataset. To enhance the precision and accuracy of the structure positioning, the slice thickness was reduced to 0.5 mm using interpolation in the C# program. All the source codes used in this manuscript can be shared by request to the corresponding author.

To assess the effect of rotational errors on dose coverage, the reference plans were copied to the rotated data sets and the dose distribution was recalculated. The dosimetric parameters such as maximum and mean dose, D99% and D95% were evaluated and compared for various rotational errors as well as the reference plans.

Dosimetric parameters were extracted from Dose Volume Histogram (DVH) in the Eclipse. There are inherent statistical uncertainties in the calculation of DVHs which are associated with the underlying discretization of structure contours. Pepin et al.^13^ addressed and assessed these uncertainties for several planning systems, including Eclipse, in their research. However, in this study where the dosimetric parameters for the same plan and similar structures between reference and rotated data were compared, it was decided not to include the inherent uncertainty present in the Eclipse DVH calculation.

The primary source of uncertainty in extracting dosimetric parameters was in the rounding errors in determining the center position of rotated spherical contours. This uncertainty was particularly significant for spherical contours with rounding error in the Z direction. This was mainly due to the restricted slice thickness and the fact that Eclipse only allows contouring on image slices and not in the space between them. To estimate the effect of this uncertainty on obtaining the dosimetric parameters, the central position of spherical volume was varied within magnitude and direction of rounding errors. Using ESAPI, spherical contours were created around the new central points and dosimetrical parameters obtained. The variations between dosimetrical parameters were used as uncertainty. This process was carried out for plans with various PTV volumes and rotational errors. The uncertainty has been included in with the results.

### 2.2 Intra‐fractional setup errors using Encompass system

The Encompass immobilization system (Avondale, PA, USA) is required as part of HyperArc for patient positioning and has been modelled in Eclipse to prevent potential collision during treatment. The Encompass system was used for positioning of non‐HyperArc stereotactic brain patients receiving a total dose of 27 Gy in three fractions. Three CBCT images were acquired during patients’ treatment: one at the start, one in the middle, and one at the end of the treatment session. Patients were repositioned according to intra‐fractional shifts measured from these CBCT images using a 6DoF couch. By retrospectively examining the CBCT images, intra‐fractional rotational setup errors during treatment were assessed. This assessment was carried out by analyzing the mid and final CBCT images obtained during treatment for a cohort of 14 patients. A total of 84 CBCT images were analyzed. This was done to assess rigidity and stability of the mask during the treatment as well as collect data on the magnitude of intrafraction setup errors. This data can be used for assessing overall precision and reliability of the treatment process and comparison made with the findings of the phantom study. The uncertainty of these results was determined by calculating the standard deviation of intrafractional errors in the analyzed images.

## 3 RESULTS

### 3.1 Uniform rotational errors around all axes

The order and direction of rotation that led to maximum displacement were consistently X(CCW) Y(CCW) Z(CCW) for all targets except those located at 4.4 and 4.5 cm from the isocenter, where the order and rotation sequence was Z(CCW) Y (CCW) X(CW). Table 1 shows the maximum displacement values for all targets with these rotational errors. For the phantom setup in this study, the displacement values do not necessarily follow a pattern of increasing with greater distance from the isocenter. Structures located at 4.4 and 4.5 cm show a higher maximum displacement compared to those at 4.9 or 5.3 cm. This discrepancy is due to the relative positioning of the targets in relation to the rotational axes. Targets located directly on the rotational axis remain unaffected by its rotation.

**TABLE 1:** Maximum displacement values for different targets and rotational errors (mm).

Rotational error (°)	Distance from the isocenter (cm)					
	1.3	1.8	4.4	4.5	4.9	5.3
0.5	0.1	0.2	0.7	0.7	0.6	0.7
1.0	0.3	0.4	1.3	1.4	1.2	1.3
1.5	0.4	0.7	2.0	2.0	1.8	2.0
2.0	0.6	0.8	2.7	2.7	2.4	2.5

**FIGURE 2:**
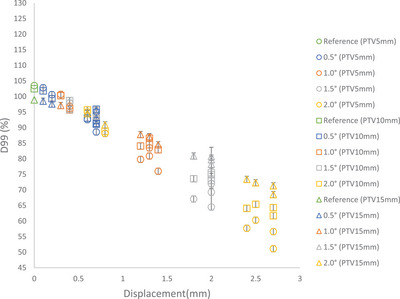
D99% versus displacement of targets for PTVs.

Figure 2 shows the relationship between D99% values and their respective displacement for all targets with different rotational errors around combined axes as previously described. As expected, a linear trend is observed between these two parameters. D99% values decrease as displacement increases. Among targets with equivalent displacement, those with smaller diameters show a more pronounced reduction in D99% values.

**FIGURE 3:**
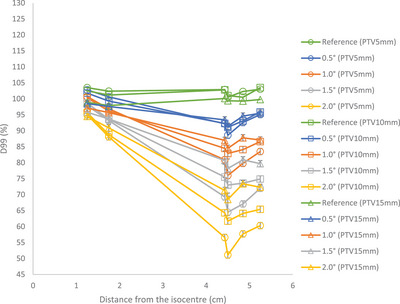
D99% versus distance from the isocenter for PTVs.

Figure 3 shows the D99% values versus distance from the isocenter for various rotational errors around the combined axes for different PTV diameters. In general, increasing rotational errors and distance from isocenter leads to a decrease in D99% values across all PTVs compared to the reference plan. However, the effect of smaller displacement that leads to higher D99% values for target located at 4.9 and 5.3 cm can be observed in Figure 3.

The susceptibility to rotational errors increases as the PTV volume decreases. For PTV5mm, a 2° rotational error results in D99% dose reduction ranging from 8.5% to 49.4%. While, for the same rotational error, the D99% dose reduction for PTV10 mm, varies between 6.8% and 39.3%, and for PTV15 mm, it ranges from 4.4% to 30.9%.

If an acceptable tolerance of 5% is established for the reduction value in D99%, only a few structures in each plan would satisfy this tolerance. For PTV5mm with a rotational error of 0.5°, only the structure located at a distance of 1.8 cm or closer with displacement value of 0.2 mm or less would meet this tolerance. With a rotational error of 1.0°, this distance reduces to only those structures located at 1.3 cm with displacement value of 0.1 mm. For PTV10mm and PTV15mm with rotational errors of 1.0° this distance is 1.8 cm. This information can be valuable in determining the maximum allowable displacement of a PTV from the isocenter while still ensuring satisfactory dose coverage.

D95% values followed a similar trend as D99% for three PTV volumes. Ideally, the D95% parameters should be close to the prescription dose. In the three reference plans, the D95% values consistently exceeded 100%. For a rotational error of 0.5°, all structures within the three PTV volumes had D95% values above 94% at various distances from the isocenter and different displacements. However, for a rotational error of 2°, D95% values declined to approximately 55%, 70%, and 75% for PTV5 mm, PTV10 mm and PTV15 mm, respectively. D95% values have been presented in Table 3 in discussion section when compared with other studies.

**FIGURE 4:**
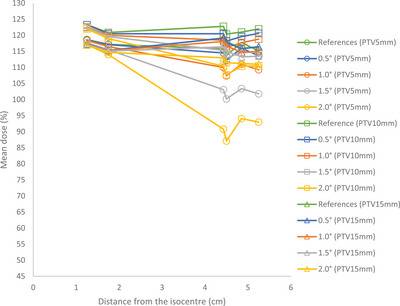
Mean dose versus distance from the isocenter for PTVs.

The maximum dose values remained consistent across all PTVs despite different rotational errors. However, the mean dose showed a declining trend as the rotational errors increased (Figure 4). With a 2° rotational error, the mean dose decreased by approximately 25%, 12%,and 6% for PTV5mm, PTV10mm, and PTV15mm, respectively.

**FIGURE 5:**
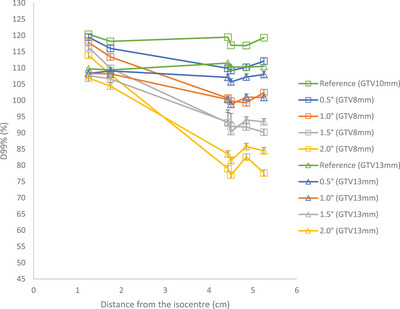
D99% versus distance from the isocenter for CTVs.

CTV structures showed similar trend to their corresponding PTV structures. Figure 5 shows the D99% values for the CTV8mm and CTV13mm that correspond to PTV10mm and PTV15mm respectively. All CTVs maintain a coverage greater than 99.3% for the D99% parameters with the rotational errors of 0.5° and 1.0°. For rotational errors of 1.5° all CTVs have D99% greater than 90% whereas for rotational errors of 2.0°, for structures located at distances greater than 1.75 cm with 2 mm displacement, the coverage dropped to around 80% to 85%.

### 3.2 Rotational errors around individual axis

Table 2 shows the reduction in the D99% parameters for rotational errors around the individual axes of X, Y, and Z. Similar to combined rotational errors, increasing the distance from the isocenter and the magnitude of the rotational errors leads to greater reduction in D99% coverage. However, the PTVs located on the rotational axes remain unaffected by the rotational errors. Consequently, the D99% reduction for PTVs located on the Z axis remained unchanged by rotational errors around Z axis (Table 2).

**TABLE 2:** Absolute percentage reduction in D99% for rotational errors around individual axes (±1% unless specified).

Rotational error (°)	Distance from isocenter (cm)																	
	PTV5mm						PTV10mm						PTV15mm					
	1.3	1.8	4.4	4.5	4.9	5.3	1.3	1.8	4.4	4.5	4.9	5.3	1.3	1.8	4.4	4.5	4.9	5.3
X = 0.5°	0.3	0.0	2.7	2.7	3.4	4.6	0.3	1.1	3.7	2.2	3.6	5.3	0.4	0.1	1.6	1.1	2.2	2.6
X = 1.0°	1.4	1.5	7.1	7.6	11.1	11.6	1.5	2.6	7.3	6.0	8.9	10.6	1.0	1.2	3.8	3.6	6.7	8.7
X = 1.5°	3.0	4.2	12.0	13.2	18.6	19.4	2.3	4.5	10.1	9.1	17.0	19.1	2.1	2.2	7.6	7.9	9.8	11.9
X = 2.0°	4.9	6.9	16.9	18.0	26.1	27.7	4.3	6.9	16.7	13.9	22.4	24.3	2.7	4.0	10.2	9.8	14.4	18.5
Y = 0.5°	0.3	0.0	2.7	2.7	3.4	4.6	0.5	1.4	8.3	7.1	4.2	4.9	0.1	0.1	4.1	3.7	2.9	2.0
Y = 1.0°	1.6	4.9	10.2	11.4	16.5	12.9	1.5	2.6	10.3	9.3	9.8	10.6	0.7	1.0	5.9	5.7	8.4	8.1
Y = 1.5°	4.1	7.4	25.9^a^	25.5^a^	27.5	22.1	2.6	4.6	18.5^b^	18.1^b^	18.4	20.5	1.4	2.3	15.2^b^	14.4^b^	13.1	12.9
Y = 2.0°	5.5	10.2	28.8	29.9	34.7	29.4	3.9	7.0	22.4	21.7	24.3	26.2	2.3	3.9	17.1	16.4	19.5	21.0
Z = 0.5°	0.1	0.0	1.2	1.6	0.1	0.0	0.0	−0.1	2.7	2.2	0.1	0.0	0.0	−0.1	1.0	1.5	0.0	−0.1
Z = 1.0°	0.1	0.0	4.6	5.2	0.1	0.0	0.0	−0.1	6.0	5.8	0.1	0.0	0.0	−0.1	3.2	3.7	0.0	−0.1
Z = 1.5°	0.0	0.0	9.4	9.4	0.0	0.0	0.0	0.0	9.3	8.9	0.0	0.0	0.0	−0.1	6.0	7.8	0.0	−0.1
Z = 2.0°	0.0	0.0	13.3	14.0	0.0	0.0	0.0	0.0	12.9	13.5	0.1	0.0	−0.1	−0.1	9.1	10.1	0.0	−0.1

^a^ Uncertainty for these values is between +1% and −6%.

^b^ Uncertainty for these values is between +1% and −3%.

Overall, the reduction in D99% around the X and Y axes were comparable across all PTV volumes in the three plans.

### 3.3 Rotational setup errors for patient treated with Encompass system

The absolute mean rotational errors for pitch, roll and yaw obtained from mid and final CBCT images were 0.17° ± 0.13°, 0.11° ± 0.10°, and 0.12° ± 0.10° respectively. The maximum rotational errors observed in this cohort of patients were 1.00°, 0.90°, and 0.80° for pitch, roll, and yaw.

## 4 DISCUSSION

In the clinical set‐up, rotational errors are not expected to have the same magnitude around all axes. However, to account for the worst‐case scenario and enable comparison with other studies, we applied uniform rotation around all three axes with the same magnitude, as described previously. These rotational errors were applied in the order and direction that resulted in the maximum displacement of the volumes. Our findings showed that the displacement of the volumes depend on its relative position to the rotational axes and does not necessary increase by increasing the distance from the isocenter. A maximum displacement of 2.7 mm was observed for targets located within a 5 cm distance from the isocenter. This finding closely corresponds to the value of 2.5 mm reported by Rojas‐Lopez.^7^

In general, increasing rotational error and distance from the isocenter led to a decrease in the mean dose, D95% and D99% parameters. However, the maximum dose remained unchanged for all volumes with different rotational errors. Moreover, smaller volumes demonstrated a higher susceptibility to dose reduction due to rotational errors. The maximum dose reduction of D99% for PTV5mm was 49.4% compared to 30.9% for PTV15 mm.

Roper et al. showed that for a 0.5° combined rotational error, the coverage of D95% for all the cases was greater than 95% which aligns with our findings of 94%. However, when a rotational error of 2° was applied, only 63% of the cases satisfied this tolerance in their study, whereas in our study, this was true for only 30% of the cases. This discrepancy may be attributed to variations in PTV volumes, different displacement values and distances from the isocenter between the two studies. It is also important to note that Roper et al. did not mention the order and direction of the rotational errors in their study. In addition, they based their data on a conventional VMAT SRS technique, while we used the HyperArc technique in our study, which potentially creates a steeper dose gradient.^10^ Roper et al. developed a formula and quantified the effect of PTV volume and distance from isocenter on D95% using multivariate regression model, analyzing 100 PTVs in 50 patients. Table 3 shows a comparison between the D95% values calculated using Roper et al.’s model and the values obtained from our study for various PTV volumes and rotational errors. In general, the absolute difference between the D95% values calculated using Roper et al.’s model and our study for a rotational error of 0.5° remained under 10% for all three volumes and structures. The largest discrepancy, around 30% and 20%, was observed for a rotational error of 2° in the PTV5mm and PTV10mm for structures located at 4.4 and 4.5 cm, respectively. Overall, good agreement was observed for larger structures with smaller rotational errors located closer to the isocenter.

**TABLE 3:** Comparison between the D95% values calculated with Roper et al.’s model and our study (%differences are absolute).

Distance from Iso (cm)		2.0°			1.0°			0.5°		
		Roper et al.	This study	%diff	Roper et al.	This study	%diff	Roper et al.	This study	%diff
PTV5mm	1.3	99.4	98.8	0.6	103.8	103.6	0.2	104.0	105.5	−1.5
	1.8	97.3	92.7	4.6	103.0	100.8	2.2	103.8	103.5	0.3
	4.4	85.8	61.1	24.7	99.2	86.2	13.0	102.6	97.1	5.5
	4.5	85.5	56.1	29.4	99.1	82.2	16.9	102.6	94.2	8.4
	4.9	84.0	62.0	22.0	98.5	84.6	13.9	102.4	97.1	5.3
	5.3	82.2	65.0	17.2	98.0	88.1	9.9	102.3	98.4	3.9
PTV10mm	1.3	104.4	100.1	4.3	104.3	103.2	1.1	104.4	105.2	−0.8
	1.8	102.2	95.3	6.9	103.6	100.3	3.3	104.1	102.7	1.4
	4.4	90.7	71.2	19.5	99.7	90.2	9.5	103.0	97.6	5.4
	4.5	90.4	68.6	21.8	99.6	88.1	11.5	103.0	96.0	7.0
	4.9	88.9	71.7	17.2	99.1	90.6	8.5	102.8	98.2	4.6
	5.3	87.2	72.6	14.6	98.5	92.3	6.2	102.6	100.2	2.4
PTV15mm	1.3	103.1	98.8	4.3	105.9	100.6	5.3	105.4	101.3	4.1
	1.8	100.9	96.2	4.7	105.2	99.3	5.9	105.2	100.2	5.0
	4.4	89.4	78.0	11.4	101.3	93.4	7.9	104.0	99.1	4.9
	4.5	89.1	75.3	13.8	101.2	91.0	10.2	104.0	97.6	6.4
	4.9	87.6	81.4	6.2	100.7	93.5	7.2	103.8	99.2	4.6
	5.3	85.9	79.4	6.5	100.1	93.0	7.1	103.7	99.8	3.9

Comparison between the study conducted by Sagawa et al.^9^ and our own investigation was challenging due to their grouping of rotational setup errors into three categories: 0°−2°, 2°−4°, and 4°. Furthermore, Sagawa et al. did not explore the correlation between dose reduction and the PTV volumes resulting from rotational setup errors. However, their study showed a 10.4% ± 10.6% decrease in dose to PTV_all_ due to rotational setup errors, based on an examination of 16 patients with multiple brain metastases (48 PTVs in total). The maximum reduction in the D95% of PTV_all_ was 37.4%, indicating a rotational setup error range of 2° to 4°. This is close to the maximum value of 46.6% observed for dose reduction in PTV5 mm, resulting from 2° combined rotational setup errors at a distance of 4.5 cm in our study. Additionally, Sagawa et al. identified a correlation between dose reduction in D99% and D95% in individual PTVs and their distance from isocenter for all rotational setup errors, which aligns with our findings.

The reduction in D99% parameters for PTV10 mm and PTV15 mm observed in this study, resulting from rotational errors, did not show strong agreement with the geometric reduction reported in Nakano et al.’s study.^6^ Overall, the magnitude of dose reduction in our study was approximately twice as large as the geometric reduction found in their study. This suggests that relying solely on geometric approximation may not be a reliable guide for predicting dose reduction.

Examining the impact of rotational errors on CTV volumes in this study is intended as a general guide and approximation. The 1 mm margin initially added to these CTVs was meant to account for uncertainties in the overall treatment process, excluding rotational errors. The effect of these uncertainties on CTV coverage has not been discussed or investigated in this study. Consequently, the dose reduction observed for CTVs with a 1 mm PTV margin in this study solely results from rotational errors, under the assumption that other uncertainties are non‐existent.

Chang^12^, ^14^ has developed a statistical model and formulated an equation to calculate the additional margin that is required to compensate CTV coverage for rotational errors in SIMT plans. Using Chang's equation, this additional margin has been calculated for the structures in this study, and the results are shown in Table 4. It worth noting that these calculated margins closely align with the maximum displacement values in Table 1. The small discrepancies between these values arise from applying different approaches. Chang's approach is designed to provide a generalized solution applicable to different target positioning scenarios, while this study is restricted to specific target positioning with maximum displacement.

**TABLE 4:** Additional PTV margin to compensate rotational errors for structures in this study calculated by Chang model (mm).

Rotational error (°)	Distance from the isocenter (cm)					
	1.3	1.8	4.4	4.5	4.9	5.3
0.5	0.0	0.1	0.3	0.3	0.4	0.4
1.0	0.1	0.2	1.0	1.1	1.2	1.3
1.5	0.2	0.4	1.8	1.9	2.1	2.3
2.0	0.4	0.7	2.7	2.7	3.0	3.3

Our results for rotational errors around each individual axis highlight the importance of target position relative to the rotational axis. While it falls beyond the scope of this study, these findings can also be extrapolated to consider the effect of rotational errors along couch, collimator and gantry axes on target dose coverage.

Investigating the clinical rotational setup errors during treatment of cranial stereotactic patients was another integral aspect of this study. The rotational setup errors obtained from CBCT images of patients treated with Encompass system in this study are consistent with the findings of other studies as shown in Table 5. Considering the mean rotational setup error values observed in this study and previous research, using a 0.5° rotational error for calculating additional margin for SIMT plans treated with Encompass system is a prudent and safe choice.

**TABLE 5:** Rotational setup errors obtained during treatment for patients treated with Encompass mask (°).

	Mean			Maximum		
Studies	Pitch (X)	Roll (Z)	Yaw (Y)	Pitch (X)	Roll (Z)	Yaw (Y)
This study	0.17 ± 0.13	0.11 ± 0.10	0.12 ± 0.10	1.00	0.90	0.80
Ohira et al.^15^	−0.1	0.0	0.0	1.0	1.0	1.0
Sagawa et al.^9^	0.29 ± 1.23	0.32 ± 1.27	0.00 ± 1.22	3.00	2.90	3.00
Amish et al.^16^ ^a^	0.25 ± 0.36	0.13 ± 0.19	0.19 ± 0.26	0.90	0.70	0.70
Amish et al.^16^ ^b^	0.18 ± 0.36	0.08 ± 0.12	0.19 ± 0.29	1.70	0.40	0.70

^a^ Between mid and initial CBCT.

^b^ Between final and mid CBCT.

Therefore, when assessing the dose coverage and maximum displacement for a 0.5° rotational error in this study, it is evident that for targets located at a distance of 4.4 cm or more from the isocenter, an additional margin of 0.7 mm is required to ensure an adequate CTV coverage.

One of the limitations of this study is its exclusive focus on the impact of rotational errors on dose coverage, neglecting the potential influence of translational setup errors and other uncertainties throughout the treatment, which can also negatively affect dose coverage. Moreover, it is important to acknowledge that the phantom used in this study was homogeneous, and thus the potential effects of tissue inhomogeneity on the dose distribution were not taken into account. Additionally, like in other studies, the rotational errors were simulated around the isocenter, which might not precisely replicate real treatment scenarios. It is important to note that in the actual treatment scenarios, rotational setup errors can happen in any direction, different magnitudes, and around any pivot point.

## 5 CONCLUSION

In our phantom study, we assessed the dosimetric effects of various rotational setup errors on SIMT HyperArc plans for the maximum displacement. Our findings were compared with previous retrospective patient studies, geometrical and statistical studies. Furthermore, we obtained and compared the intrafractional rotational setup errors for patients undergoing treatment with Encompass system.

It was concluded that using a 0.5° rotational setup error for patient treated with Encompass is a safe choice for calculating the additional margin required to ensure adequate CTV coverage. This information combined with maximum displacement and dosimetric parameters values, can serve as a guide for determining the optimal PTV margin.

Overall, our study contributes to the knowledge and understanding of the effects of rotational setup errors on SIMT plans, helping to improve treatment accuracy and optimize patient outcomes.

## AUTHOR CONTRIBUTIONS

Samane Golmakani designed the project and wrote the manuscript and gathered the outcome data. Andrew N. McGrath provided all the required scripting for the project, contributed to the project's design, and wrote the scripting section of the manuscript, in addition to proofreading the rest of the document. Timothy J. Williams provided valuable support for the project and offered proofreading for the manuscript.

## CONFLICT OF INTEREST STATEMENT

The authors declare that there is no conflict of interest that could be perceived as biasing the impartiality of the reported research.

## Data Availability

The data that support the findings of this study are available from the corresponding author upon reasonable request.
